# Evaluating the Tensile Properties of Aluminum Foundry Alloys through Reference Castings—A Review

**DOI:** 10.3390/ma10091011

**Published:** 2017-08-30

**Authors:** A.R. Anilchandra, Lars Arnberg, Franco Bonollo, Elena Fiorese, Giulio Timelli

**Affiliations:** 1 Department of Mechanical Engineering, BMS College of Engineering, Bengaluru 560019, India; 2 Department of Materials Science and Engineering, Norwegian University of Science and Technology (NTNU), N-7491 Trondheim, Norway; lars.arnberg@ntnu.no; 3 Department of Management and Engineering (DTG), University of Padova, Stradella S. Nicola, 3 I-36100 Vicenza, Italy; bonollo@gest.unipd.it (F.B.); elena.fiorese@hotmail.it (E.F.); timelli@gest.unipd.it (G.T.)

**Keywords:** reference casting, aluminum alloy, foundry, tensile property, microstructure

## Abstract

The tensile properties of an alloy can be exploited if detrimental defects and imperfections of the casting are minimized and the microstructural characteristics are optimized through several strategies that involve die design, process management and metal treatments. This paper presents an analysis and comparison of the salient characteristics of the reference dies proposed in the literature, both in the field of pressure and gravity die-casting. The specimens produced with these reference dies, called separately poured specimens, are effective tools for the evaluation and comparison of the tensile and physical behaviors of Al-Si casting alloys. Some of the findings of the present paper have been recently developed in the frame of the European StaCast project whose results are complemented here with some more recent outcomes and a comprehensive analysis and discussion.

## 1. Introduction

The foundry industry has constantly tried to address the challenge of producing high quality and cost effective castings as the final applications demand stringent conditions. There have been constant efforts to minimize defects and imperfections in the castings and to optimize the microstructure, keeping in mind the main variables related to the employed alloy, the initial melt quality and the process conditions.

From the design viewpoint, the knowledge of tensile properties of cast products is a relevant topic, which is not fully covered by existing international standards, except for the newly introduced document CEN/TR 16748:2014 [1]. As a matter of fact, the European standards on foundry Al alloys (EN 1676 and EN 1706) provide interesting information, which should be integrated with more recent studies. Particularly, the EN 1706 standard [2] specifies the chemical composition limits for Al casting alloys and their tensile properties. As specified in [2], the yield strength (YS), the ultimate tensile strength (UTS) and the elongation to fracture (EL%) in as-die-cast condition are, respectively, 140 MPa, 240 MPa and less than 1% for the most high-pressure die-cast (HPDC) alloys, while these are around 90 MPa, 170 MPa and in the range 1–2.5% for the main gravity die-cast (GDC) alloys. Hence, the EN 1706 standard reports minimal and conservative values of the tensile properties, by considering that the average content of defects and imperfections in Al alloy castings could worsen their static strength. Recently, a consortium called StaCast [3], comprised of various institutions from European countries, was constituted and successfully proposed in [1], which should be used together with the existing standards for the evaluation of Al alloy tensile properties. In general, the CEN/TR shows higher values of tensile properties for Al alloys based on studies that optimize and improve die parameters, by consequently minimizing defects and imperfections and by improving microstructure in the castings.

The variables affecting the tensile strength of cast Al alloys are certainly well articulated and complex, involving casting and testing issues. Indeed, the static strength of foundry alloy components mainly depends on alloy composition, casting conditions, heat treatment, geometry of separately poured specimens and test conditions. Since the effects of alloying and heat treatment do not belong to the scope of the work, this review presents analysis and comparison of the tensile properties measured on the separately poured specimens obtained using dies and process parameters that were recently made part of [1] in order to explore the tensile strength of die cast Al-foundry alloys. The most popular Al foundry alloys were identified through a questionnaire: about 60 foundries, which cover a consistent percentage of the European production, have answered to the questionnaire [3]. It was found that the most common alloy category is comprised of Al-Si based alloys, both for HPDC and GDC processes. For instance, StaCast consortium results revealed that the AlSi9Cu3(Fe) alloy is used by around 59% of European foundries, while both AlSi7Mg0.3 and AlSi12Cu1(Fe) alloys are used by around 35% [3]. Moreover, 31% of foundries employ AlSi11Cu2(Fe) and a percentage lower than 30% employs other alloys, such as AlSi12(Fe) [3]. The focus of this paper is on the alloys that have large diffusion with the aim of proposing a significant contribution both for scientific and industrial fields. The alloys that have been studied in this work are usually employed for manufacturing housings, thin-wall parts and safety components [3]. Characteristics of reference dies for testing tensile behavior of foundry alloys are described in Section 2 of the present paper. The tensile properties have been reported and discussed in Section 3 for exploring the maximum static strength of foundry alloys with the aim of proposing additional tools for selecting material and designing components. Microstructure features and casting defects have also been mentioned for the alloys investigated. For more details about these aspects, the authors have recently published a work, in which the frequency of different kinds of defects is analyzed both for HPDC and GDC [4].

## 2. Reference Castings for Testing Tensile Behavior

A reference die is a permanent mold, designed according to the state-of-the-art methodologies and made of steel or cast iron, suitable for the evaluation of the static strength of a cast alloy. The geometry of such a die varies in accordance with the applied kind of process, i.e., HPDC or GDC. The specimens manufactured using such a reference die are called *separately poured specimens*.

As for process, the casting parameters affecting the quality of components were deeply reviewed in previous works for HPDC [5] and GDC [6]. It was observed that, by modifying the existing standard permanent molds, higher tensile properties could be obtained from conventional Al foundry alloys. Numerical simulation studies have been quite effective in understanding the distribution of porosity during melt flow and optimizing die parameters [7]. Regarding mechanical test conditions, capacity of machine, sensor system, cross-head speed, data elaboration, time between manufacturing and test, and testing temperature affect the behavior of castings. Tensile specimens can be round or flat, machined or not, and are characterized by a specific geometry (i.e., gauge length, gauge width and radius). In this work, the focus is on two different kinds of reference dies for testing tensile behavior of Al alloys, both in HPDC and GDC.

### 2.1. Reference Castings for High-Pressure Die-Cast Al-Si Alloys

**Figure 1:**
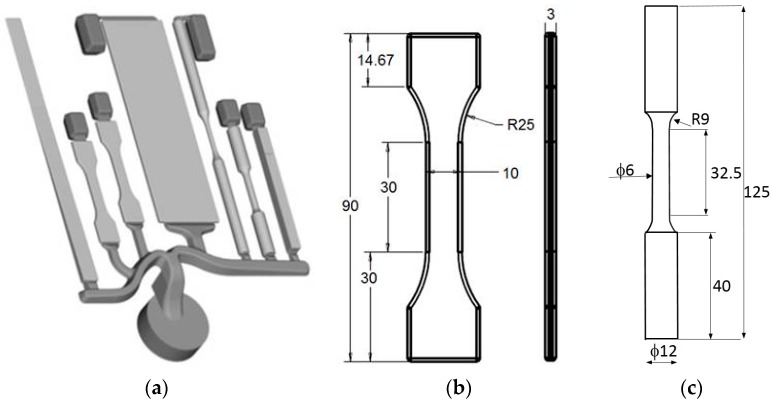
(**a**) Reference casting #1 for high pressure die casting; (**b**) flat; and (**c**) round tensile specimens (dimensions in mm) [8,9].

The static strength of HPDC Al-Si alloys can be evaluated by the reference die designed, built and tested in the frame of the NADIA Project (New Automotive components Designed for and manufactured by Intelligent processing of light Alloys, Contract No. 026563-2, 2006–2010). The reference casting #1, shown in Figure 1a, is suitable for various kinds of characterization, as it has round fatigue and stress-corrosion bars, corrosion-Erichsen test plate, fluidity and Charpy test appendices, besides the flat tensile bars. The dimensions of round and flat tensile specimens are shown in Figure 1b,c [8,9].

**Figure 2:**
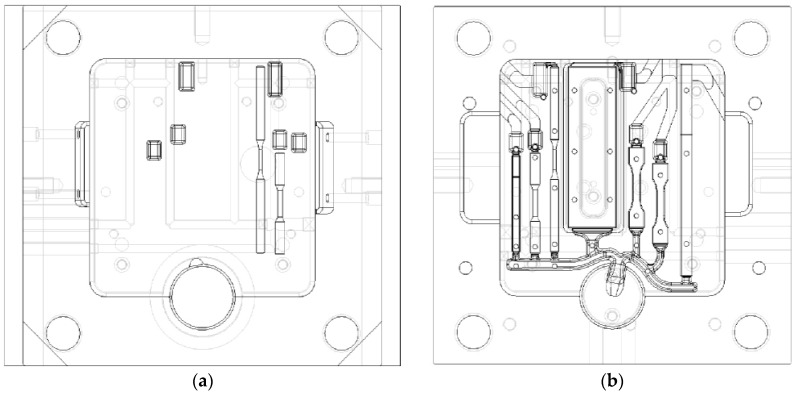
Layout of the die for diecasting of specimens: (**a**) fixed side and (**b**) ejector side [9].

**Figure 3:**
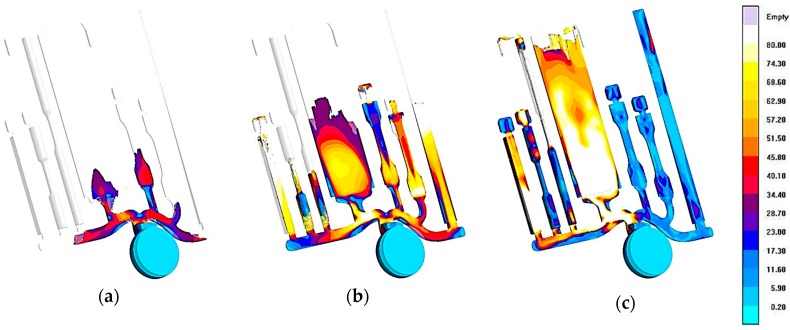
Calculated melt velocity at (**a**) 44%; (**b**) 66%; and (**c**) 92% of die filling. The color code indicates velocity in m s^−1^ [9].

This reference die is made by two AISI H11 parts, a fixed side and ejector side, and is shown in Figure 2 [9]. A detailed description of the features and the optimization of the die design, developed through numerical simulation, is given in [9]. The die was carefully designed to obtain a uniform molten metal front and a favorable thermal evolution inside the die cavity. Figure 3 shows some results of the numerical simulation of the filling process, which provides evidence of the melt velocity distribution inside the die cavity at three different percentages of the die cavity filling [9]. Further experimental details, such as the mold filling time and the initial melt quality, can be accessed from [9].

Timelli et al. [10,11] successfully used this reference die to determine the tensile properties and the microstructural characteristics of diecast AlSi9Cu3(Fe) alloy. It was observed that the tensile specimens from the reference casting #1 show significantly higher tensile strength and elongation as compared to machined specimens from the same casting. This is related to heterogeneity in the microstructure from the periphery to the center of the cross section of the die-cast specimens. It is reported that there exists a surface layer of about 1.3-mm thick in the tensile bars that is free of porosity [10,11], which contributes to higher mechanical properties in die-cast specimens if compared to machined ones. 

The reference casting #1 has been also adopted for the experimental validation of an analytical method for explaining and predicting the quality of castings, by means of the root means square plunger acceleration and the plunger speed extracted from the plunger displacement curve [12,13].

**Figure 4:**
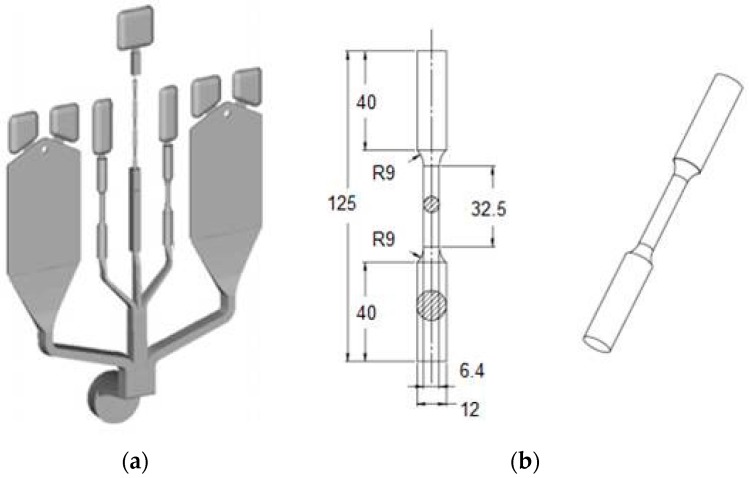
(**a**) HPDC reference casting #2; (**b**) round tensile bar (dimensions in mm) [14].

The mechanical behavior of Al foundry alloys can also be estimated by means of the reference die (Figure 4a) designed, built and tested by HYDRO in cooperation with NTNU (University of Science and Technology, Trondheim, Norway). The dimensions of the cylindrical tensile test specimens are shown in Figure 4b [14]. Some previous works [15,16] demonstrated that the tensile specimens show higher porosity in the grip section as compared to the gauge section. Moreover, the findings indicate that the central region of the grip section solidifies later than both the surface region and the gauge section (see Figure 3c). Similar observations to those made for the tensile specimens from the reference casting #1 have been proposed by Timelli et al. for the reference casting #2 [11]. 

### 2.2. Reference Castings for Gravity Die-Cast Al-Si Alloys

Gravity casting, being the simplest and conventional route of casting, has few disadvantages such as entrained air, bubbles and oxide bi-films. The design of sprue, runner and gating system is critical in minimizing defects and imperfections. It can be achieved by critical calculations, like those proposed by Campbell [17]. 

**Figure 5:**
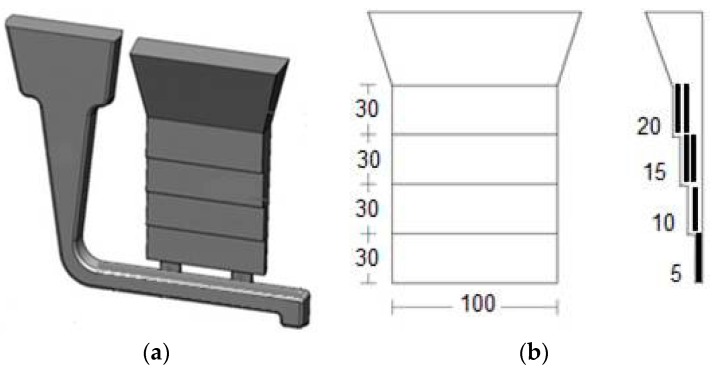
(**a**) Reference casting #3 for gravity die casting; (**b**) sectioning scheme for mechanical property testing (dimensions in mm) [20,22].

**Figure 6:**
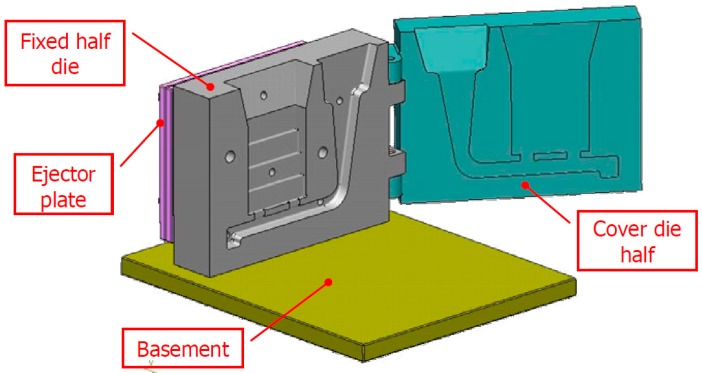
Layout of the die [20].

The ASTM B108 gives dimensions and details of a standard round tensile specimen specifically designed for GDC [18], which is also called a Stahl mold. Efforts made by researchers and foundries to adopt the Stahl mold for obtaining the best mechanical properties of cast alloys are reviewed in [6]. The limitation of the Stahl mold was that it has well-defined dimensions in terms of gauge length and thickness, with the consequence that it was not allowed to study different solidification rates. To overcome this limitation, the Aluminum Association developed a step configuration of the die with variable thickness [19]. The design proposed by Grosselle et al. [20], shown in Figure 5 and Figure 6 as reference casting #3, consists of four steps varying from 5 to 20 mm, from which flat tensile bars can be machined as per the ASTM B557 [21] with gauge length, width and thickness of 30, 10 and 3 mm, respectively.

The step casting was gated from the bottom of the thinnest step, while the riser over the casting ensures a good feeding. This configuration allows for obtaining a range of cooling rates and consequently different microstructural scales in the casting [20]. As shown in Figure 6, the two-part die is split along a vertical joint line passing through the pouring basin. To facilitate assembly and mutual location, the die halves are hinged. The dimension of the whole die is 310 × 250 × 115 mm^3^ and the thicknesses of the two die halves are 45 and 75 mm, respectively [20,22]. 

Variations of the step design have been recently studied to improve the quality of test specimens obtained. It is worth noticing the optimization of the step mold design proposed by Timelli et al. for Mg alloys, which could be probably used for other foundry alloys [23].

**Figure 7:**
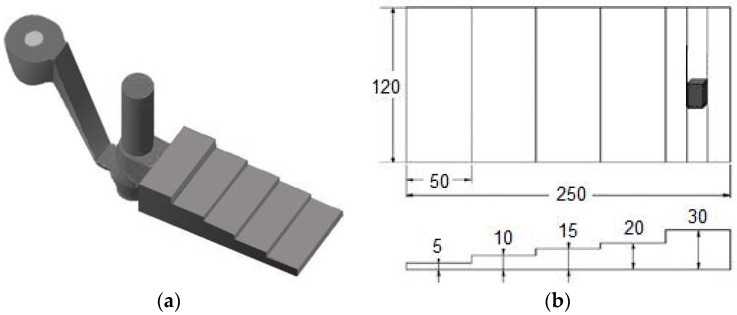
(**a**) Reference casting #4 for gravity die casting; (**b**) sectioning scheme for mechanical property testing (dimensions in mm) [24,25].

The mechanical behavior of GDC Al-Si alloys can be evaluated by another step reference die designed, built and tested by NTNU (University of Science and Technology, Trondheim, 7491, Norway) in cooperation with SINTEF (Trondheim, Norway). As shown in Figure 7, the reference casting #4 has five steps, 250 mm length, 120 mm width and thickness ranging from 5 to 30 mm [24,25]. Round tensile bars can be machined from steps with 30, 20, 15 and 10 mm thickness, while flat bars from the 5 mm step. The round bars have 36 mm gauge length and 10 mm total diameter, while the flat bars have 32 mm gauge length, 10 mm total width and 5 mm thickness.

## 3. Results on the Expected Tensile Strength of Al-Si Alloys Cast in Permanent Mold

The expected tensile strength is the mechanical behavior that can be achieved by Al-Si alloys, cast in reference dies with state-of-the-art knowledge on die design, process management and alloy treatments properly applied to minimize casting defects and imperfections and to improve the microstructure. The expected tensile strength of Al-Si alloys was estimated by means of tensile testing performed on specimens obtained through the above-described dies.

### 3.1. Expected Tensile Strength of High-Pressure Die-Cast Alloys

The tensile strength of HPDC alloys was been obtained through reference casting #1 [1,8,9]. Table 1 collects the chemical composition of the alloys tested, which have been selected based on the considerations made in the Introduction. The addition of iron permits avoiding soldering phenomenon due to high velocity and pressure typical of the HPDC process [26].

The alloys, supplied as commercial ingots, were melted in a 300 kg crucible in a gas-fired furnace set up at (800 ± 10) °C and maintained at this temperature for at least 3 h. The temperature of the melt was then gradually decreased by following the furnace inertia up to (690 ± 5) °C. The molten metal was degassed with Ar for 15 min. Since the initial metal quality can deeply affect the castability of an alloy [27,28] and the final properties of castings, the quality of the materials used in the experimental campaigns was estimated by Foseco H-Alspek to measure hydrogen level and by reduced pressure test (RPT) to measure bi-film index. The hydrogen content was lower than 0.15 mL/100 g Al during the entire experimental campaigns while the bi-film indexes were in the range between 10 and 18 mm. A bi-film index is generally used to determine the molten metal quality: the higher the bi-film index is, the lower the tensile test values and the higher the scatter are [25,29]. Periodically, the molten metal was manually skimmed with a coated paddle.

Cast-to-shape specimens were produced using HPDC reference casting #1 in a cold chamber die-casting machine with a locking force of 2.9 MN. The nominal plunger velocity was 0.2 m/s for the first phase and 2.7 m/s for the filling phase; a pressure of 40 MPa was applied once the die cavity was full. The optimal experimental conditions at steady-state, which guarantee a high quality of castings, were found to be: pouring temperature 690 °C, melt velocity at in-gates 51 m/s and filling time 9.7 ms. Indeed, the process parameters influence defect content and microstructure of castings, as it has been deeply studied in [13]. The cycle time was approximately 45 s.

The weight of the Al alloy die-casting was 0.9 kg, including the runners, gating and overflow system. About 15 castings were scrapped after the start-up, in order to reach a quasi-steady-state temperature in the shot chamber and the die. Oil circulation channels in the die served to stabilize the temperature (at ~230 °C). The melt was transferred in 18 s from the holding furnace and poured into the shot sleeve by means of a coated ladle. The fill fraction of the shot chamber, with 70 mm inner diameter, was 0.28.

The surface finish of samples was adequately accurate to avoid machining, and only some excess flash along the parting line of the die was manually removed. The tensile tests have been done on a tensile testing machine. The crosshead speed used was 2 mm/min and the strain was measured using a 25-mm extensometer. Experimental data have been collected and processed to provide yield stress (YS or 0.2% proof stress), ultimate tensile strength (UTS) and elongation to fracture (%EL). At least 10 tensile tests were conducted for each condition. The specimens were maintained at room temperature for five months before testing. Table 2 summarizes the tensile strength of the alloys tested. 

Tensile properties of the round specimens obtained through AlSi9Cu3(Fe) alloy can be compared with those achieved in a previous study [10] using the same die. The values of mechanical properties are in good agreement, by highlighting effectiveness of the proposed die in evaluating the properties of castings.

Properties reported in Table 2 can be also compared with those obtained in another study [30], which used a different geometry called “one bar casting” with a single large feeding channel on one extremity of the specimen, thus to have a coaxial inflow with the specimen. Moreover, a large feeder was added on the other extremity of the specimen. The resultant as-cast tensile bar was cylindrical with gauge length 30 mm, total length 96 mm and gauge diameter 8 mm. For the AlSi10Cu3(Fe) alloy, the following properties were achieved: 275 MPa UTS, 150 MPa YS and 2.1% EL. Besides its effectiveness, another advantage of the proposed die is that multiple specimens for tensile, fatigue, impact, corrosion and stress-corrosion testing can be prepared from a single casting.

**Figure 8:**
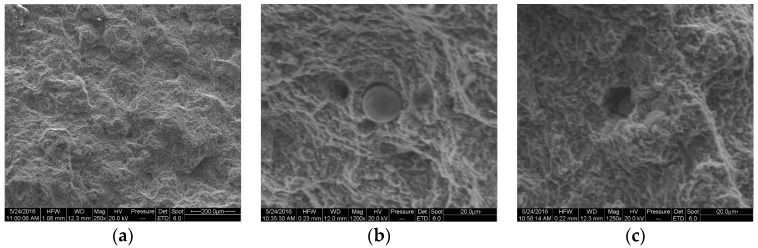
SEM micrographs of the fracture surface of the round specimen obtained through reference die #1 and AlSi12Cu1(Fe) alloy: (**a**) low magnification image; (**b**) cold shot and (**c**) micro-porosity. These defects are small thanks to the optimized geometry of the die.

This reference die was carefully designed and optimized to maximize the quality of castings, by reducing the scrap percentage and the presence of defects and imperfections. With the aim of demonstrating this statement, a typical fracture surface under the scanning electron microscope (SEM) is reported in Figure 8a, and some sample defects detected in the specimen at higher magnification are shown in Figure 8b,c. These defects are detrimental for tensile properties and could cause premature failure of castings. Nevertheless, they are small thanks to the optimized geometry of reference die and the optimal experimental conditions adopted. More on casting defects and their effect on mechanical properties could be found in compendium [4]. 

### 3.2. Expected Tensile Strength of Gravity Die-Cast Alloys

The tensile strength of GDC alloys was evaluated through reference castings #3 and #4. Table 3 collects the composition of the alloys tested. These alloys were selected based on the popular Al foundry alloys identified through a questionnaire [3] (as described in the Introduction of this work) and are frequently used for manufacturing automotive components, such as cylinder heads, wheels and carter. The chemical composition of the alloys was properly chosen for improving the final properties of castings. For instance, the addition of Cu permits enhancing strength and workability of an alloy, while the presence of Fe improves wear resistance [22,26].

The alloys, supplied as commercial ingots, were melted in 70 kg electric resistance furnace set up at (720 ± 10) °C and maintained at this temperature. The molten metal was then degassed with Ar for 15 min. The hydrogen content of the melt in the holding furnace was analyzed by hydrogen analyzer, and it showed values lower than 0.1 mL/100 g Al during the entire experimental campaign. Periodically, the molten metal was manually skimmed with a coated paddle. Sr modification was carried out as well as Ti grain refining; lower mechanical properties can be expected without these metal treatments. Castings were produced using reference castings #3 and #4. The temperature of the die was maintained at around 300 °C by means of oil circulation channels, and about three castings were scrapped after the start-up to reach a quasi-steady-state temperature in the die. A ceramic filter with a pore size of 10 ppi was used. The optimal filling time was 5–6 s.

Flat tensile test bars with rectangular cross section were drawn from each step, in the middle zones of the castings and the dimensions were maintained as per the ASTM-B557 standard. The tensile tests have been done on a tensile testing machine with crosshead speed of 1.5 mm/min. The strain was measured using a 25-mm extensometer.

Table 4 summarizes the tensile strength of the investigated alloys. Several specimens (around 10) from each step were tested.

**Figure 9:**
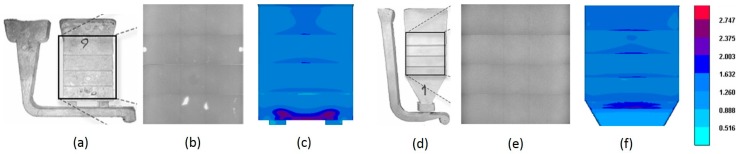
(**a**) step casting produced with reference die #3; (**b**) defects observed under X-ray scan; (**c**) distribution of micro-porosity due to shrinkage predicted by magma-simulation; (**d**) modified step casting; (**e**) improved quality of casting as observed under X-ray scan; (**f**) distribution of micro-porosity percentage (%) as indicated by the color code [23].

The AlSi7Mg0.3 alloy is equivalent to the popular A356 cast alloy and the minimum standard tensile values of this alloy reported in the literature are 145 MPa (UTS) and 3% EL. The AlSi6Cu4 is equivalent to A319 alloy whose minimum strength values reported in the literature are 186 MPa (UTS) and 2.5% EL, measured using the popular ASTM B-108 mold [18]. Considering the values reported in [1,18], the values of Table 4 for reference die #3 are disappointing and offer scope for the improvement in the mold design. Generally, low elongation to fracture is related to the presence of diffused microscopic casting defects such as oxides, porosity, etc. For similar alloy composition, Akhtar et al. [25] observed higher ductility using reference die #4. The comparison between reference dies #3 and #4 indicates that the die configuration is an important parameter in assessing the tensile potential of an alloy as much as the melt quality and the pouring conditions. Timelli et al. [23] noted that by modifying the runner and gating systems in reference die #3, the amount of casting defects could be minimized. This was shown in both experimental and numerical simulation studies. The modified die design is represented in Figure 9. However, the study was limited to microstructural characterization of magnesium alloys and needs to be extended to aluminum alloys. By means of numerical simulation techniques, Wang et al. [31] found that the gauge section of the standard Stahl mold (standard ASTM B-108) showed higher porosity (about 3%) compared to the AA Step mold (less than 1%) in an AlSi7Mg alloy. This comparison confirmed the statement of Singworth and Kuhn [32] that the Stahl mold could not produce better mechanical properties than the Step mold, due to higher micro-porosity in the gauge section on account of micro-shrinkage. However, the AA Step mold is different from reference die #3 in terms of in-gate and runner design. To the best of the authors’ knowledge, there is no research available about the comparative studies between the Stahl mold and the modified Step mold.

**Figure 10:**
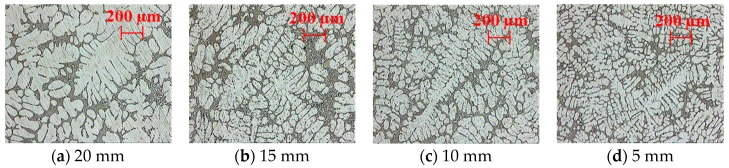
Microstructure of the AlSi7Mg0.3 alloy with decreasing step thickness from (**a**–**d**). These micrographs are related to interior of the casting (see Figure 5b).

Figure 10 shows the microstructure of the AlSi7Mg0.3 alloy as a function of step thickness. It is worth noticing that the geometry of the proposed reference die permits accurately evaluating the mechanical properties of the alloy with thickness variations. As the step thickness reduces, the cooling rates are higher and secondary dendritic arm spacing (SDAS) is lower (Figure 10d), resulting in improved mechanical properties. Average SDAS of each step has been measured and was 45.4 µm, 32.4 µm, 30.1 µm and 25.0 µm by reducing the thickness (from Figure 10a–d). Percentage of porosity has also been estimated and was 0.65, 0.42, 0.22 and 0.05 by reducing the step thickness. Similar observations were made in the work of Grosselle et al. [20,22] with reference die #3, which is concomitant with observations made using reference die #4 for similar alloy composition [24,25].

## 4. Conclusions

This review work indicates that the tensile properties of high-pressure and gravity die-cast Al-Si alloys are better than what has been previously estimated by the existing standards. The improved mechanical properties are due to the minimized casting defects and imperfections, and optimized microstructure of specimens obtained through the reference castings proposed.

With the aim of precisely estimating the mechanical behavior of aluminum alloys, reference dies for HPDC and GDC should be chosen and tensile testing conditions should be standardized. For the HPDC process, the reference die #1 developed in the frame of NADIA Project can be used, since it simultaneously provides specimens for tensile, fatigue, impact, corrosion and stress-corrosion testing. The step casting obtained using reference die #4 for GDC permits evaluating the mechanical properties as a function of thickness variations, which strongly affect cooling rate and hence the resulting microstructure. However, reference die #3 needs modification and could be used after optimizing as proposed for Mg alloys.

The knowledge of the tensile strength of foundry alloys, which can be obtained through reference castings, will give a relevant contribution to material selection and design approach, by allowing to choose the best solution for the envisaged application. This knowledge will significantly help the foundries to reduce production costs by minimizing scrap with a consequent improvement in the competitive edge.

## Figures and Tables

**Table 1:** Chemical composition of the investigated high pressure die cast Al-Si alloys (wt.%).

Alloy	Si	Fe	Cu	Mn	Mg	Cr	Ni	Zn	Pb	Sn	Ti	Al
AlSi9Cu3(Fe)	8.227	0.799	2.825	0.261	0.252	0.083	0.081	0.895	0.083	0.026	0.041	bal.
AlSi11Cu2(Fe)	10.895	0.889	1.746	0.219	0.224	0.082	0.084	1.274	0.089	0.029	0.047	bal.
AlSi12Cu1(Fe)	10.510	0.721	0.941	0.232	0.242	0.045	0.080	0.354	0.055	0.025	0.038	bal.

**Table 2:** Tensile strength properties of the investigated Al-Si HPDC alloys obtained through reference die #1. Flat specimens with 3 mm thickness and round specimens with 6 mm diameter.

Alloy	Type of Specimen	UTS (MPa)	YS (MPa)	EL (%)
AlSi9Cu3(Fe)	Flat	309 ± 6	163 ± 1	3.6 ± 0.3
	Round	342 ± 8	168 ± 6	5.1 ± 0.4
AlSi11Cu2(Fe)	Flat	312 ± 2	153 ± 1	3.5 ± 0.1
	Round	342 ± 7	158 ± 3	5.5 ± 0.7
AlSi12Cu1(Fe)	Flat	283 ± 2	137 ± 1	3.5 ± 0.1
	Round	315 ± 7	131 ± 2	7.1 ± 0.5

**Table 3:** Chemical composition of the investigated gravity die cast Al-Si alloys (wt. %).

Alloy	Si	Fe	Cu	Mn	Mg	Ni	Zn	Ti	Al
AlSi7Mg0.3	6.5	0.1	0.002	0.007	0.3	0.003	0.006	0.1	bal.
AlSi6Cu4	6.0	1.0	4.0	0.5	0.1	0.3	1.0	0.2	bal.

**Table 4:** Average tensile strength of Al-Si GDC alloys evaluated by means of two different reference die.

Alloy	Step Thickness (mm)	Reference Die #3		Reference Die #4	
		UTS (MPa)	EL (%)	UTS (MPa)	EL (%)
AlSi7Mg0.3	5	181	2.3	194 ± 2	9.5 ± 1
	10	172	1.7	182 ± 3	7.1 ± 0.5
	15	182	2.3	174 ± 1	5.6 ± 0.3
	20	167	1.8	166 ± 2	4.4 ± 0.2
	30	-	-	161 ± 3	3.2 ± 0.6
AlSi6Cu4	5	203	0.9	-	-
	10	207	1.0	-	-
	15	194	0.8	-	-
	20	188	0.7	-	-
